# Fat mass and obesity-associated gene (FTO) rs9939609 (A/T) polymorphism and food preference in obese people with low-calorie intake and non-obese individuals with high-calorie intake

**DOI:** 10.1186/s40795-023-00804-y

**Published:** 2023-12-06

**Authors:** Mehran Rahimlou, Bijan Ghobadian, Ali Ramezani, Ehsan Hejazi, Saeideh Mazloomzadeh, Jalal Hejazi

**Affiliations:** 1 Department of Nutrition, School of Medicine, Zanjan University of Medical Sciences, P.O. Box 4517713433, Zanjan, Iran; 2 Department of Internal Medicine, School of Medicine, Vali-e-Asr Hospital, Zanjan University of Medical Sciences, Zanjan, Iran; 3 Biotechnology Departments, School of Pharmacy, Zanjan University of Medical Sciences, Zanjan, Iran; 4 Departments of Clinical Nutrition and Dietetics, Faculty of Nutrition and Food Technology, National Nutrition and Food Technology Research Institute, Shahid Beheshti University of Medical Sciences, Tehran, Iran; 5 Rajaie Cardiovascular Medical and Research Center, Iran University of Medical Sciences, Tehran, Iran

**Keywords:** Food preferences, FTO rs9939609, Obesity, Polymorphism, Sweet

## Abstract

The purpose of this study was to assess the connection between FTO rs9939609 (A/T) polymorphism and food preference. The study included 77 participants, 36 of whom were obese and had a low-calorie intake, and 41 non-obese participants with a high-calorie intake. Using a food frequency questionnaire (FFQ), the researchers calculated sweet and fatty food propensity scores. Genomic DNA was extracted from a peripheral blood sample from all participants, and FTO rs9939609 (A/T) polymorphism was assessed using standard methods. The study found no significant differences between the two groups in terms of sweet food preference (15.64 ± 10.53 in obese groups vs. 14.72 ± 7.95 in the non-obese group, p = 0.711) and fatty food preference (16.81 ± 8.84 vs. 17.27 ± 8.75; p = 0.833). Additionally, the study did not find any significant correlation between FTO rs9939609 (A/T) polymorphism and sweet and fatty food preferences in the fully adjusted models (p > 0.05). Therefore, the results of this study do not support the hypothesis of different food preferences.

**Supplementary Information:**

The online version contains supplementary material available at 10.1186/s40795-023-00804-y.

## Introduction

Obesity is a major health issue worldwide, with its incidence doubling since 1980 in over 70 countries and continuing to rise in most other nations [1]. It is known as the mother of all diseases due to its significant association with conditions such as type 2 diabetes mellitus(T2DM), fatty liver disease, hypertension, myocardial infarction, stroke, dementia, osteoarthritis, obstructive sleep apnea, and many cancers [2].

The number of calories consumed and Basal Metabolic Rate (BMR) are among the most important factors contributing to obesity. However, recent attention has been given to whether controlling calorie intake is an effective solution to prevent the obesity epidemic [3]. This question arises from the observation of thin people who consume high calories but do not gain weight, and obese people who consume low calories but do not experience significant weight loss. Studying this group of obese and non-obese individuals can help us better understand the etiology of obesity. Some researchers suggest that factors such as food behavior, genetic polymorphisms, and hormonal factors may be effective in addition to the concept of calories alone [3, 4].

Appetitive behavior is significantly influenced by genome-wide effects [5–7], and single-gene hypomorphic mutations that code for parts of canonical pathways controlling metabolic rate and expenditure (such as LEP, LEPR, and MC4R) can cause severe hyperphagia and early-onset obesity [8, 9]. Despite adhering to the same food habits, individuals may move towards a range of body weights. Genetics has been shown to be responsible for over 40% of the observed differences between people in terms of BMI [10]. According to some studies, genetic polymorphisms can impact the development of obesity through three pathways: the central nervous system (CNS), adipogenesis, and energy balance regulation pathways [11–13]. The fat mass and obesity-associated (FTO) gene, located on chromosome 16q12.2 in intron 1, is a crucial gene linked to obesity pathogenesis. It regulates adipogenesis by adjusting alternative splicing through m^6^A demethylation, which triggers mitotic clonal expansion during early adipogenesis [14–16]. Overexpression of the FTO gene has been found to induce adipogenesis by targeting nearby genes involved in energy balance regulation and white adipocyte development [17]. In a meta-analysis study by Peng et al., it is reported that FTO gene rs9939609 polymorphism is significantly related to obesity risk [18].

Numerous studies have indicated that the FTO A-allele may be linked to specific food preferences, particularly those that are high in calories. For instance, Tanofsky-kraff and colleagues conducted a cross-sectional study involving children and adolescents aged 6–19 years old, which revealed that the FTO gene rs9939609 polymorphism (T/A substitution) heightens the preference for high-fat foods and increases consumption of calorie-dense foods [19].

There have been numerous studies indicating a strong correlation between the preference for fatty and sweet foods and obesity. It appears that the liking of these high-calorie foods is more likely to cause obesity than the other way around. However, some studies have found no significant differences in fatty and sweet food preferences between obese and non-obese individuals, and some have even suggested that people with obesity may have a lower inclination towards sweet foods [20].

Given the significance of genetic polymorphisms in food preference and the potential impact of food preference on the development of obesity, this study aimed to examine the connection between FTO polymorphism and food preference in two groups: obese individuals with low-calorie intake and non-obese individuals with high-calorie intake.

## Materials and methods

### Participants and research plan

This case-control study was conducted between June 2020 and September 2021 at the specialized nutrition clinic of Zanjan University of Medical Sciences. The study included two groups: obese individuals with low-calorie intake and non-obese individuals with high-calorie intake. The sample size for the study was determined to be 70 subjects, with 35 participants in both the case and control groups, based on Yang et al.‘s research [15]. The study was conducted in compliance with the Declaration of Helsinki and the protocol of the research was approved by the Ethics Committee of the Zanjan University of Medical Sciences (Ethics Code: IR.ZUMS.REC.1399.089) and the Ethics Committee of the National Institute for Medical Research Development (Ethics Code: IR.NIMAD.REC.l397.539). Before the data collection, participants were explained the aims and methodology of the study and then were asked to sign a written informed consent letter.The study was approved by the Prior to the commencement of the study, written informed consent was obtained from all participants.

The case group consisted of 36 subjects who were classified as obese with a BMI greater than 30. These individuals were recognized by their friends and family as having a low appetite, and their average daily calorie intake was determined to be less than 50% of the required calories based on the Mifflin St. Jeor equation, which was calculated using a 3-day, 24-hour recall method [21]). The control group consisted of 41 participants who had a BMI of less than 25 kg/m2. These individuals were recognized by their peers as having a high appetite, and their average daily energy intake was estimated to be more than 50% of the required calories.

Individuals who had been on a weight reduction diet within the past year, had a disease that could impact weight (such as diabetes, thyroid disorders, cancer, polycystic ovary syndrome, etc.), or were taking medications that could affect weight (such as corticosteroids) were not included in the study.

### General questionnaires

All participants provided sociodemographic information, including their age, gender, ethnicity, and history of disease or drug use. The participants’ height and weight were measured while they were fasting and wearing light clothing, and Body mass index (BMI) was calculated by dividing the weight (kg) by height squared (m^2^).

### Dietary intake assessment

The researchers used a semi-quantitative food frequency questionnaire (FFQ) consisting of 147 food items to collect dietary data. Trained dietitians asked participants about their intake frequency for each food item they consumed over the past year, with responses recorded as daily, weekly, or monthly intakes, and then converted to weekly intakes. Previous studies have evaluated and confirmed the validity and reliability of this questionnaire for assessing the dietary intake of the Iranian population [22]. Individuals with a daily energy intake greater than 4300 or less than 670 kcal were excluded. The FFQ consisted of 23 fatty food items (such as fried potatoes, canned tuna, haslet, hamburgers, sausages, rumen, Khash, animal tongue and brain, whole-fat milk, whole-fat yogurt, full-fat cheese, cream, butter, margarine, fried onion, oil, mayonnaise and mayonnaise-based products, peanut butter, fried eggs, chocolate- or nut-based spread, nuts and seeds, chocolate-based candies, cake/pudding/cookies) and 19 sweet items (including fruit juices, carbonated sugar-sweetened drinks, sugar-sweetened drinks not carbonated, flavored milk, sugar added breakfast cereals, ice cream, raisin, fruit compote, sugar, honey, gaz (a Persian candy), sohan (a Persian candy), candy, halva, jam, cake/pudding/cookies, sweetened coffee and sweetened tea). To calculate the sweet and fatty food propensity scores, the consumption frequency of each sweet and fatty food was divided by the consumption frequency of all foods in the FFQ and multiplied by 100. This resulted in a continuous variable with scores ranging from 0 to 100% indicating the preference for sweet and fatty food. [23, 24].

### DNA extraction and genotyping

To conduct genetic evaluations, 10 ccs of venous blood were drawn from all participants and placed in vacuum collection tubes containing EDTA. Genomic DNA was extracted from a peripheral blood sample of each participant using a DNA extraction kit according to the manufacturer’s instructions (Viragen, Tehran, Iran). The quality of the extracted DNA was assessed by measuring OD260/OD280 and concentration using a nanodrop instrument (Eppendorf, Germany). The FTO gene’s single nucleotide polymorphism (SNP) rs9939609(A/T) was directly genotyped by amplifying the rs9939609(A/T) region of the gene using gene-specific oligonucleotide primers. Genotypes for the FTO rs9939609 polymorphism (TT/AT/AA) were determined via amplification refractory mutation system polymerase chain reaction (ARMS-PCR). Table 1 S and Table 2 S display the primer sequences and PCR conditions. Each PCR was initially conducted in a 25 µL mixture, which included 300 ng of genomic DNA, 10 pM of each primer, and 25 µl of 2× Master Mix (Fermentas, USA) containing Taq DNA polymerase, MgCl2, dNTPs, and reaction buffers. The PCR products were then analyzed through 2% agarose gel electrophoresis and viewed under UV light. Next, 25 µl of each PCR product with the forward primer was sent for direct sequencing (Pishgam, Tehran, Iran). Sequencing was performed using the Sanger method on an ABI 3730 sequencer. The resulting sequences were analyzed using BLAST, Clustal X2, and Chromas V2.4 software.

### Statistical analysis

The Kolmogorov-Smirnov test was utilized to assess whether the variables were normally distributed. As the data was found to be normally distributed, a t-test for independent samples was employed to compare the means between the two groups. Logistic regression was conducted to compute the odds ratio, with age and sex included in the model as confounding variables. The Hardy-Weinberg Equilibrium (HWE) was performed to evaluate the genes variant among cases and controls, and the chi-square test was used to compare the allelic frequency distributions between different variants. IBM-SPSS software version 26 was utilized to analyze all of the data, and a p-value of less than 0.05 was considered statistically significant.

## Results

The study included 77 adult participants, with a mean age of 32.64 ± 8.06 years in the case group (participants with obesity) and 29.36 ± 9.82 years in the control group (non-obese group), which was not significantly different (P = 0.174). The male participants in the case group accounted for 19.4%, while in the control group, it was 36.58% (P = 0.097). The mean weight of participants in the case group was 92.96 ± 12.86 kg, which was significantly different from the control group’s mean weight of 56.17 ± 8.87 kg (P < 0.001). The case group consumed 1161.35 ± 357.61 kcal from their diet, while the non-obese group consumed 3129.90 ± 1002.01 kcal (Table 1).

**Table 1:** Participants’ characteristics

Variables	Obese group (n = 36)	Non-obese group (n = 41)	***P***_value
Age (years)	32.64 ± 8.06	29.36 ± 9.82	0.174
Sex (Female%)	29 (80%)	26 (63%)	0.097
Height (cm)	160.43 ± 8.51	166.12 ± 9.52	0.008
Weight (Kg)	92.96 ± 12.86	56.17 ± 8.87	< 0.001
BMI (kg/m^2^)	36.12 ± 4.22	20.37 ± 2.96	< 0.001
WC (cm)	108.46 ± 10.45	79.37 ± 9.34	< 0.001
Hip (cm)	119.75 ± 11.55	95.37 ± 8.04	< 0.001
Calories (Kcal)	1161.35 ± 357.61	3129.90 ± 1002.01	< 0.001

BMI: Body Mass Index; WC: waist circumference

Table 2 shows a comparison between sweet and fatty food preference scores among the obese and non-obese groups. The mean sweet preference score in the obese group was 15.64 ± 10.53, which was not statistically different from the non-obese group’s mean score of 14.72 ± 7.95 (P = 0.711). Similarly, there were no significant differences between the obese and non-obese groups in terms of fat preference score (16.81 ± 8.84 vs. 17.27 ± 8.75; P = 0.833).

**Table 2:** Food preference to sweet and fatty foods among obese and non-obese subjects

Variables^1^	Total (n = 77)	Case (n = 36)	Control (n = 41)	***P***-value^2^
Sweet preference Score	15.15 ± 9.10	15.64 ± 10.53	14.72 ± 7.95	0.711
Fatty preference Score	17.07 ± 8.72	16.81 ± 8.84	17.27 ± 8.75	0.833

^1^Values are presented as mean ± SD, ^2^Calculated by independent sample t test

Table 3 displays a comparison of food preferences between participants with AT/AA and TT genotypes. The results show that there were no significant differences between the two groups in terms of sweet preference scores (14.15 ± 7.64 vs. 14.49 ± 8.09; P = 0.877) and fatty food preference scores (16.74 ± 9.04 vs. 17.53 ± 9.36; P = 0.762).

**Table 3:** Food preference to sweet and fatty foods among subjects with AT/AA and TT genotypes

Variables^1^	AT/AA	TT	***P***-value^2^
Sweet preference Score	14.15 ± 7.64	14.49 ± 8.09	0.877
Fatty preference Score	16.74 ± 9.04	17.53 ± 9.36	0.762

^1^Values are presented as mean ± SD, ^2^Calculated by independent sample t test

Table 3 also presents the association between rs9939609 polymorphism and fat and sweet preference scores. The results indicate that there was no significant association between FTO polymorphism and sweet preference score in both the crude (P = 0.985) and fully adjusted models (P = 0.59). Similarly, there was no significant association between FTO polymorphism and fatty food preference scores in both the crude (P = 0.91) and fully adjusted models (p = 0.81).

Table 4 shows the association between propensity scores for sweet and fatty food and the risk of obesity between obese group and non obese group. The results indicate that there was no significant association between sweet and fatty food preference and the risk of obesity.

**Table 4:** Association between propensity scores for sweet and fatty food and the risk of obesity

	Non-obese group	Obese group	***P*** ^1^
Sweet preference Score	1	0.98 (0.93, 1.04)	0.69
Fatty food preference Score	1	1.007(0.95,1.06)	0.81

Association was reported as OR (95% CI), Calculated by logistic regression

## Discussion

Obesity is a chronic disease that involves multiple factors in its development. Recent studies have shown that the idea of calorie intake alone cannot fully explain the occurrence of obesity, and that various factors, including genetic polymorphisms and eating behaviors, play a role [25, 26]. The findings of this study indicate that there was a significant difference in the frequency of rs9939609 polymorphism between obese individuals with low-calorie intake and non-obese individuals with high-calorie intake. However, there were no significant differences between these two groups in terms of their preference for sweet and fatty foods. Additionally, there was no significant association between rs9939609 polymorphism and food preference for sweet and fatty foods.

Consistent with our results, Hasselbalch et al. conducted a population-based study on 756 healthy adult twin pairs to examine the relationship between FTO rs9939609 polymorphism and habitual dietary intake. They discovered that there was no correlation between this SNP and habitual dietary intake [27].

In contrast to our findings, Daya et al. conducted a case-control study on obese and non-obese participants in Jakarta to assess the relationship between FTO gene rs9939609 polymorphism and the risk of obesity and preference for fatty foods. They found that individuals with AT/AA genotypes had a 5.98 times higher intake of dietary fat compared to those with TT genotype [11]. Susmiati et al. also conducted a study on adolescent girls in Indonesia and reported that those with AT/AA genotypes consumed more fried foods and had lower fruit consumption than those with TT genotype [28]. Cecil et al. demonstrated that the FTO gene rs9939609 polymorphism plays a crucial role in appetite regulation, leading to a hyperphagic phenotype and a preference for energy-dense foods [16]. Furthermore, Tanofsky et al. conducted a cross-sectional study and observed that individuals with this polymorphism had a greater tendency to consume energy-dense foods, resulting in a significant increase in their weight [19].

In a cross-sectional study, Brunkwall et al. investigated the association between rs9939609 polymorphism of the FTO gene and food preferences in healthy adults. They found that participants who carried the A-allele had a greater tendency to consume biscuits and pastries but had lower consumption of soft drinks [29]. The differences observed between the results of these studies and ours could be explained by the variations in the populations studied. In our study, the obese group had limited calorie intake despite being obese. In contrast, previous studies have shown that obese individuals tend to consume higher calorie diets, particularly from fatty and sweet foods [30, 31].

The incidence of obesity worldwide has been linked to high-fat and energy-dense diets, as evidenced by numerous studies [32]. Obese individuals have been found to have a stronger preference for fats, which are a concentrated source of energy with rewarding post-ingestive effects [32–34]. The theory that obese people have a sweet tooth has also been extensively researched, with expectations of preferences for sweet meals in this population. However, the outcomes of these studies have been inconsistent. Some studies have shown that obese individuals appreciate sweetness to the same degree or even less than non-obese individuals, and enjoyment of meals does not significantly differ between obese and non-obese people [35–37]. These findings are consistent with our own.

The present study had several strengths.To our knowledge, this study is the first to examine the relationship between FTO rs9939609 SNP and the preference for sweet and fatty foods in obese individuals with low-calorie intake and non-obese individuals with high-calorie intake. Also, we set strict inclusion criteria to minimize the effect of confounding factors. However, there were some limitations to this study. The self-reported methods used to assess both calorie intake and sweet and fatty food propensity scores are subject to reporting bias, which is a limitation. Additionally, the relatively small sample size of this study is another limitation, and our findings should be confirmed by larger studies in this population.

## Conclusion

According to the findings of this study, there were no significant differences in the preference for sweet and fatty foods between the obese group with low-calorie intake and the non-obese group with high-calorie intake. Furthermore, we did not observe a significant correlation between FTO rs9939609 SNP and sweet and fatty food preferences. However, further research is required to validate the results of this study.

### Electronic supplementary material

Below is the link to the electronic supplementary material.

Supplementary Material 1

Supplementary Material 2

## Data Availability

The datasets generated and/or analysed during the current study are available in the Figshare repository, [PERSISTENT WEB LINK: https://figshare.com/articles/dataset/Supporting_Information_file_for_manuscript_entitled_b_FTO_rs9939609_A_T_polymorphism_and_food_preference_b_/24417856 ]
