# Supplementary material for: Fat mass and obesity-associated gene (FTO) rs9939609 (A/T) polymorphism and food preference in obese people with low-calorie intake and non-obese individuals with high-calorie intake
Source: BMC Nutr. 2023 Dec 6;9:143. doi: 10.1186/s40795-023-00804-y (PMC10698998; doi:10.1186/s40795-023-00804-y)
Supplement: Supplementary file 1 — Supplementary Material 1 [file 40795_2023_804_MOESM1_ESM.docx]

Table 1S: Temperature properties of primers

| **Polymorphism** | **Direction** | **Primer sequence** | **OD^*^** | **Tm^**^** |
| --- | --- | --- | --- | --- |
| Rs9939609 | Forward | 5-TTGATACACTGCCCCTACCC-3 | 6/5 | 60 |
|  | Reverse | 5-TCCCAAAGTCCTGGAAACAC-3 | 6/4 | 58 |

* Optical Density

** Melting Temperature
