# Supplementary material for: Fat mass and obesity-associated gene (FTO) rs9939609 (A/T) polymorphism and food preference in obese people with low-calorie intake and non-obese individuals with high-calorie intake
Source: BMC Nutr. 2023 Dec 6;9:143. doi: 10.1186/s40795-023-00804-y (PMC10698998; doi:10.1186/s40795-023-00804-y)
Supplement: Supplementary file 2 — Supplementary Material 2 [file 40795_2023_804_MOESM2_ESM.docx]

Table 2S: PCR temperature program

| Repetition | Steps | Temperature | Time |
| --- | --- | --- | --- |
| 1 | Primary denaturation | 95°C | 5 min |
| 40 | Denaturation | 95°C | 30 second |
|  | Binding the primer to the template | 58°C | 30 second |
|  | Elongation | 72°C | 30 second |
| 1 | Final elongation | 72°C | 7 minutes |
